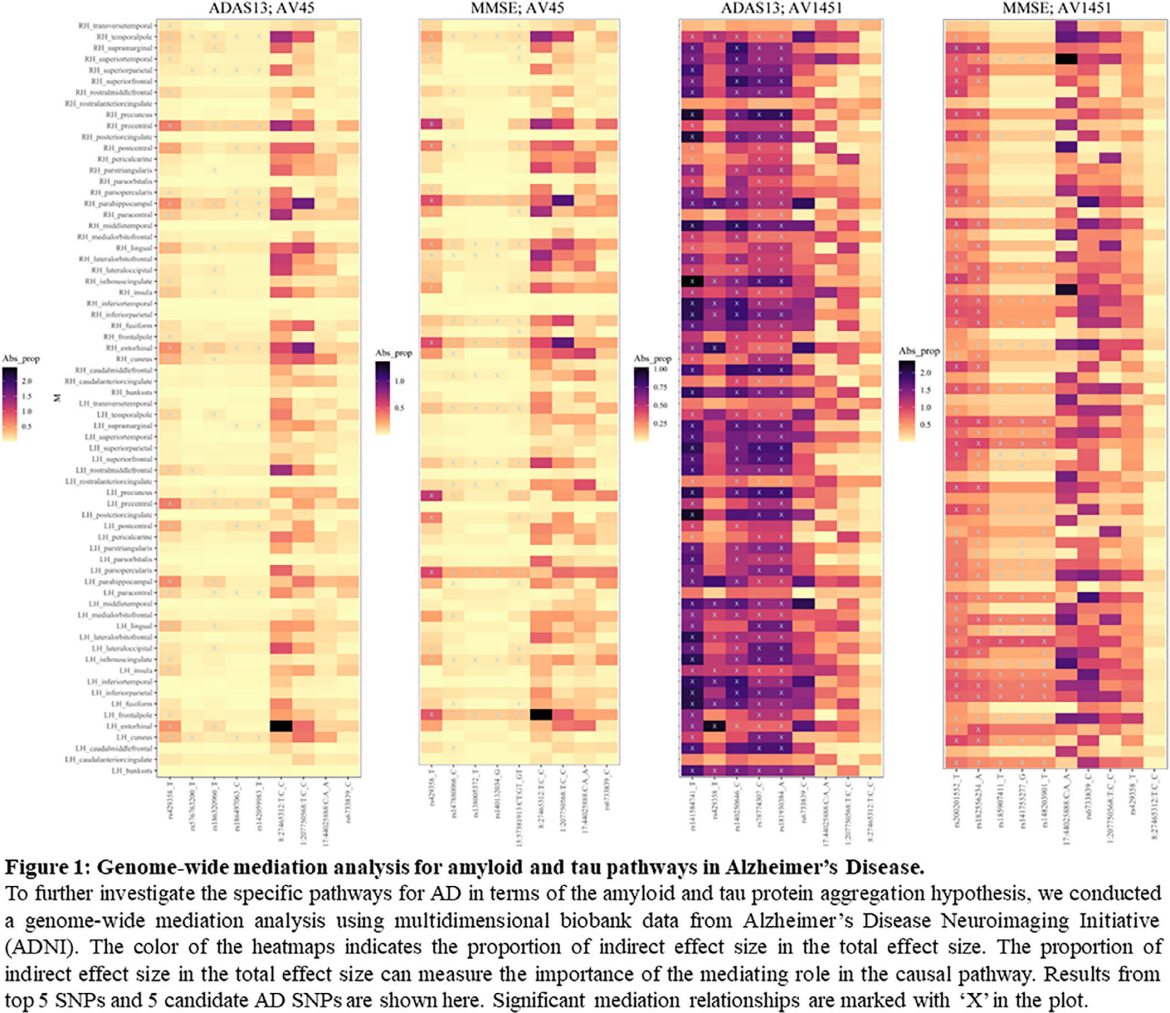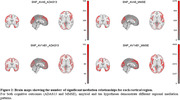# Genome‐wide mediation analysis reveals amyloid and tau imaging genetics patterns in Alzheimer’s Disease

**DOI:** 10.1002/alz.089710

**Published:** 2025-01-09

**Authors:** Shizhuo Mu, Saima Rathore, Jingxuan Bao, Shu Yang, Basavaraj Hooli, Li Shen, for the ADNI

**Affiliations:** ^1^ University of Pennsylvania, Philadelphia, PA USA; ^2^ Eli Lilly and Company, Indianapolis, IN USA

## Abstract

**Background:**

Alzheimer’s disease (AD) is a progressive neurological disorder with an unclear cause, and its amyloid and tau hypotheses need deeper investigation to understand their link to genetic variants and AD outcomes. To bridge this gap, we conduct a mediation analysis using the genotyping, amyloid and tau imaging, and cognitive data from Alzheimer’s Disease Neuroimaging Initiative (ADNI) to delineate specific mediation pathways from genetic variants such as single nucleotide polymorphisms (SNPs), to regional amyloid/tau protein aggregation in the brain, and to cognitive outcomes.

**Method:**

ADNI provides genotyped data and harmonized phenotypes. We further imputed, harmonized, annotated the genotyping data. Post‐quality control and subject matching resulted in 1,051 individuals for amyloid and 525 for tau imaging measurements. We first conducted genome‐wide association study (GWAS) on 5,302,064 variants to prioritize SNPs significantly associated with cognitive scores. Subsequently, we employed a mediation model to explore how regional amyloid and tau imaging measures mediate the genetic effects on cognitive scores. Specifically, we used prioritized SNPs from GWAS as exposure, two cognitive scores (ADAS‐Cog13 and MMSE) as outcomes, and amyloid and tau imaging measures from 68 cortical regions as mediators. Additionally, we contrasted results from GWAS‐identified SNPs with five candidate AD SNPs.

**Result:**

We assessed amyloid and tau aggregation in 68 cortical brain regions as mediators for SNP effects on cognitive scores; see Figure 1 for top findings. After Bonferroni correction, we found 9,551, 34,402, 5,636, and 193,002 mediation relationships for SNP‐amyloid‐ADAS13, SNP‐amyloid‐MMSE, SNP‐tau‐ADAS13, and SNP‐tau‐MMSE respectively. Tau aggregation demonstrated a broader impact on AD across cortical regions compared to amyloid. Although 4 of the candidate AD SNPs showed no signal, one of them, rs429358, demonstrated strong amyloid and tau mediated genetic effects on cognitive outcomes. Our results also showed that amyloid and tau hypotheses have different regional mediation patterns (Figure 2). For example, bankstss region only influences the tau pathway, while caudal anterior cingulate only affects the amyloid pathway.

**Conclusion:**

Our genome‐wide mediation analysis has identified top cortical regions and SNPs associated with amyloid‐ and tau‐mediated cognitive declines in AD. These results yield new insights into AD etiology linking genetic determinants, cellular hallmarks, and phenotypic outcomes.